
JOURNAL INFORMATION
==============================
NLM Title Abbreviation: ScientificWorldJournal
Journal ID: ScientificWorldJournal
NLM Journal ID: 101131163
Journal ID: TSWJ

The Scientific World Journal

ISSN: 2356-6140
EISSN: 1537-744X
Publisher: Wiley

ARTICLE INFORMATION
==============================
PMCID: PMC6084512
PMID: 30147608
DOI: 10.1100/tsw.2001.224
Article ID: 963431
Article version: 1
Subjects: Short Report

Necrotic Neurodegeneration in Caenorhabditis Elegans

Xu Keli
Gerstbrein Beate
Driscoll Monica
Department of Molecular Biology and Biochemistry, Rutgers University, 604 Allison Road, Piscataway NJ 08855, USA
Publication date: 2001
Electronic publication date: 2001 Dec 12
Volume: 1
First page: 129
Last page: 129
Copyright: Copyright © 2001 Keli Xu et al.
Copyright year: 2001
License: This is an open access article distributed under the Creative Commons Attribution License, which permits unrestricted use, distribution, and reproduction in any medium, provided the original work is properly cited.
License URL: https://creativecommons.org/licenses/by/3.0/

==============================
Miami *Nature Biotechnology* Short Reports
*TheScientificWorld* (2001) 1 (S3), 129SR
ISSN 1532-2246; DOI 10.1100/tsw.2001.224

### NECROTIC NEURODEGENERATION IN *CAENORHABDITIS ELEGANS*

Keli Xu, Beate Gerstbrein, and Monica Driscoll

Department of Molecular Biology and Biochemistry, Rutgers University, 604 Allison Road, Piscataway NJ 08855

Necrosis plays a central role in neuronal injury associated with stroke and ischemia. Unlike apoptotic cell death, little is understood of molecular mechanisms of necrosis. The two distinct forms of cell death, necrosis and apoptosis, are easily distinguishable by morphological characteristics in the nematode *Caenorhabditis elegans*. At the level of light microscopy, necrotic cells appear as swollen vacuoles several times larger than normal cells; apoptotic cells become compacted and raised. Under the electron microscope the first visible step in necrotic-like death is the involution of the plasma membrane, followed by the appearance of membrane whorls [1]. Later, the electron density of the cytoplasm is reduced, the cell swells and cytoplasmic vacuoles arise. The chromatin forms aggregates and becomes fragmented. Organelles swell and finally lyse. The dead cell is removed by phagocytosis [2]. The key characterized initiators and regulators of apoptotic cell death do not influence the initiation or progression of necrotic cell death [2].

A variety of different insults can initiate necrotic-like neurodegeneration in *C. elegans* and lead to similar morphological changes in the affected cells. Necrosis-inducing genes include dominant alleles of the *mec-4* and *deg-1* degenerins which encode ion-channel subunits with similarity to mammalian epithelial $Na^+$-ion channels (ENaC) [3, 4, 5], a dominant allele of the *deg-3* acetylcholine receptor $\alpha$-subunit [6], and a transgenically expressed constitutively activated mutant of the G-protein subunit $G\alpha_S$ [7]. That channel hyperactivating mutations induce necrosis is reminiscent of the initiation of excitotoxic cell death in humans.

In our lab we study necrotic neurodegeneration with a focus on channel-hyperactivating mutations in the *mec-4* gene as the death-initiating event. The MEC-4 protein is expressed in the six touch neurons in *C. elegans*, where it participates in the formation of mechanically gated ion channels and is involved in the signal transduction of mechanical stimuli. The dominant *mec-4(d*) allele encodes a hyperactive channel that leads to necrotic demise of the touch receptor neurons. We established a behavioral assay that allowed us to isolate suppressor mutations that block *mec-4(d)*-induced neurodegeneration. Ectopic expression of *mec-4(d)* in the ventral nerve cord leads to the appearance of a number of swelling and degenerating cells visible as vacuoles during the larval stage L1. As a consequence of this cell death, worms are severely paralyzed. We mutagenized 45.000 haploid genomes and isolated 24 worms with reconstituted locomotion. Such strains harbor candidate suppressors of *mec-4(d)*-induced neurodegeneration. Ten of the suppressors proved to be alleles of *mec-6*, which is a known suppressor of ectopically expressed *mec-4(d).* The other loci define several previously unknown genes involved in *mec-4(d)*-induced cell death.

We have identified one strong suppressor locus as calreticulin [8]. Calreticulin is a $Ca^{2+}$ binding protein of the ER that plays critical roles in chaperone function and in maintenance of $Ca^{2+}$ homeostasis. We have also shown that mutations in $Ca^{2+}$ release channels *itr-1* (IP3 receptor) and *unc-68* (ryanodine receptor) also significantly suppress *mec-4(d)*-induced neurodegeneration. Our data suggest release of ER $Ca^{2+}$ stores is a critical step in necrotic cell death in *C. elegans*. Since ER $Ca^{2+}$ release has been implicated in excitotoxic cell death, our data also supports that necrotic death mechanisms may be conserved from nematodes to humans.

We are currently investigating the function and identity of the remaining death suppressor alleles to decipher molecular mechanisms of necrotic-like cell death in the nematode.

**REFERENCES.**

1. Hall, D.H., Gu, G., Garcia-Anoveros, J., Gong, L., Chalfie, M., and Driscoll, M. (1997) Neuropathology of degenerative cell death in *Caenorhabditis elegans*. J. Neurosci. 17, 1033-1045
2. Chung, S., Gumienny, T.L., Hengartner, M.O., and Driscoll, M. (2000) A common set of engulfment genes mediates removal of both apoptotic and necrotic cell corpses in *C. elegans*. Nat. Cell Biol. 2, 931-937
3. Driscoll, M. and Chalfie, M. (1991) The *mec-4* gene is a member of a family of *Caenorhabditis elegans* genes that can mutate to induce neuronal degeneration. Nature 349, 588-593
4. Chalfie, M. and Wolinsky, E. (1990) The identification and suppression of inherited neurodegeneration in *Caenorhabditis elegans*. Nature 345, 410-416
5. Tavernarakis, N., Shreffler, W., Wang, S., and Driscoll, M. (1997) *unc-8*, a DEG/ENaC family member, encodes a subunit of a candidate mechanically gated channel that modulates *C. elegans* locomotion. Neuron 18, 107-119
6. Treinin, M. and Chalfie, M. (1995) A mutated acetylcholine receptor subunit causes neuronal degeneration in *C. elegans*. Neuron 14, 871-877
7. Korswagen, H.C., Park, J.H., Ohshima, Y., and Plasterk, R.H. (1997) An activating mutation in a *Caenorhabditis elegans* $G_S$ protein induces neural degeneration. Genes Dev. 11, 1493-1503
8. Xu, K., Tavernarakis, N., and Driscoll, M. (2001) in preparation.
